# Serotonergic Modulation of Neurovascular Transmission: A Focus on Prejunctional 5-HT Receptors/Mechanisms

**DOI:** 10.3390/biomedicines11071864

**Published:** 2023-06-29

**Authors:** Abimael González-Hernández, Bruno A. Marichal-Cancino, Antoinette MaassenVanDenBrink, Carlos M. Villalón

**Affiliations:** 1 Instituto de Neurobiología, Universidad Nacional Autónoma de México, Boulevard Juriquilla 3001, Queretaro 76230, Mexico; abimaelgh@comunidad.unam.mx; 2 Departamento de Fisiología y Farmacología, Universidad Autónoma de Aguascalientes, Mexico City 20100, Mexico; bruno.marichal@edu.uaa.mx; 3 Division of Vascular Medicine and Pharmacology, Department of Internal Medicine, Erasmus MC University Medical Center, 3000 CA Rotterdam, The Netherlands; a.vanharen-maassenvandenbrink@erasmusmc.nl; 4 Departamento de Farmacobiología, Cinvestav-Coapa, Calzada de los Tenorios 235, Colonia Granjas-Coapa, Delegación Tlalpan, Mexico City 14330, Mexico

**Keywords:** serotonin, CGRP, blood pressure, migraine, hypertension

## Abstract

5-Hydroxytryptamine (5-HT), or serotonin, plays a crucial role as a neuromodulator and/or neurotransmitter of several nervous system functions. Its actions are complex, and depend on multiple factors, including the type of effector or receptor activated. Briefly, 5-HT can activate: (i) metabotropic (G-protein-coupled) receptors to promote inhibition (5-HT_1_, 5-HT_5_) or activation (5-HT_4_, 5-HT_6_, 5-HT_7_) of adenylate cyclase, as well as activation (5-HT_2_) of phospholipase C; and (ii) ionotropic receptor (5-HT_3_), a ligand-gated Na^+^/K^+^ channel. Regarding blood pressure regulation (and beyond the intricacy of central 5-HT effects), this monoamine also exerts direct postjunctional (on vascular smooth muscle and endothelium) or indirect prejunctional (on autonomic and sensory perivascular nerves) effects. At the prejunctional level, 5-HT can facilitate or preclude the release of autonomic (e.g., noradrenaline and acetylcholine) or sensory (e.g., calcitonin gene-related peptide) neurotransmitters facilitating hypertensive or hypotensive effects. Hence, we cannot formulate a specific impact of 5-HT on blood pressure level, since an increase or decrease in neurotransmitter release would be favoured, depending on the type of prejunctional receptor involved. This review summarizes and discusses the current knowledge on the prejunctional mechanisms involved in blood pressure regulation by 5-HT and its impact on some vascular-related diseases.

## 1. Introduction

Among all biogenic monoamines, serotonin (5-hydroxytryptamine; 5-HT) stands out for its complex effects, the participation of a wide variety of receptors (which include the 5-HT_1_, 5-HT_2_, 5-HT_3_, 5-HT_4_, 5-HT_5_, 5-HT_6_, and 5-HT_7_ receptors), and its extensive distribution in vertebrates and invertebrates [1]. In mammals, 5-HT is mainly synthesised in enterochromaffin cells (~90%) and in serotonergic neurons of the brain (1–2%) [2]. Indeed, this monoamine is predominantly found in platelets, enterochromaffin cells and in the central nervous system (CNS), but in many cases, its physiological role remains elusive [1,3]. Fortunately, with the progressive development of agonists and antagonists that act selectively on 5-HT receptors, many functions of 5-HT in the CNS and in the periphery have been discovered [1,3].

### 1.1. A Summary on 5-HT Receptors

This review will not document historical aspects of 5-HT research, discovery or 5-HT receptors. However, published research on the mechanisms involved in the effects of 5-HT (even long before its identification as 5-HT) has accumulated over 130 years [1,3,4,5].

As summarized in Table 1, with the conjunction of structural, transductional, and operational (pharmacological) criteria, 5-HT receptors have been classified into seven receptor types (5-HT_1_-5-HT_7_) that can be grouped into: (i) six metabotropic (G-protein-coupled) receptors, namely: the 5-HT_1_ (further subdivided into the 5-HT_1A_, 5-HT_1B_, 5-HT_1D_, 5-ht_1e_ and 5-HT_1F_ subtypes), 5-HT_2_ (further subdivided into the 5-HT_2A_, 5-HT_2B_ and 5-HT_2C_ subtypes), 5-HT_4_, 5-HT_5_ (further subdivided into the 5-HT_5A_ and 5-ht_5B_ subtypes), 5-HT_6_ and 5-HT_7_ receptor types; and (ii) one ligand-gated ion channel represented by the ionotropic 5-HT_3_ receptor type [1,3,4,5]. The corresponding subtypes of the 5-HT_1_, 5-HT_2,_ and 5-HT_5_ receptor types share similar structural and transductional properties, but display very different pharmacological profiles.

Some agonists and antagonists employed to identify the pharmacological profile of each 5-HT receptor type are shown in Table 1. As previously established [1,3,4,5], the pharmacological identification of a specific 5-HT receptor type is based on the application of (i) inclusion criteria (i.e., selective agonists for this receptor mimic the effects of 5-HT, while selective antagonists for this receptor produce a blockade of the effects of 5-HT and the corresponding agonist); and (ii) exclusion criteria (i.e., agonists and antagonists for the other 5-HT receptors—and sometimes even for receptors unrelated to 5-HT—are inactive) (see Table 1).

This knowledge (i) has helped to establish the role of 5-HT receptors in several diseases, including anxiety, depression, schizophrenia, drug addiction, cardiovascular pathologies (e.g., systemic, pulmonary and portal hypertension), cardiac disorders, migraine, etc.; and (ii) has led to the development of agonists and antagonists at 5-HT receptors for the therapeutic treatment of these—and other—diseases [1,3,4,5,6,7,8].

### 1.2. An Overview of the Effects of 5-HT on the Cardiovascular System

As previously described in other reviews dealing with 5-HT and the cardiovascular system [3,6,7,8,9], the cardiovascular effects of 5-HT are complex and include bradycardia/tachycardia, hypotension/hypertension, and vasodilatation/vasoconstriction. This complexity of effects is due to (i) the capability of 5-HT to interact at various levels, including the heart and blood vessels, as well as the central and peripheral (autonomic and sensory) nervous systems; and (ii) the involvement of serotonin 5-HT_1_, 5-HT_2_, 5-HT_3_, 5-HT_4_, 5-HT_5A,_ and 5-HT_7_ receptors, as well as a tyramine-like action or unidentified mechanisms, depending on the species and the experimental conditions [3,6,7,8,9]. Interestingly, the 5-HT_6_ receptor is not involved in the cardiovascular effects of 5-HT [3,8].

### 1.3. The Specific Interactions of 5-HT at Peripheral and Central Levels to Induce Cardiovascular Effects

#### 1.3.1. Sensory Afferents

Overall, an intravenous (i.v.) bolus injection of 5-HT in anaesthetised animals results in a reflex bradycardia and hypotension by stimulating 5-HT_3_ receptors on vagal sensory afferents [3]. These neuronal 5-HT_3_ receptors were identified using selective agonists and antagonists (see Table 1).

#### 1.3.2. Sympathetic Ganglia

It has been shown that i.v. 5-HT can stimulate and/or inhibit the sympathetic ganglia producing stimulation or inhibition of the sympathetic drive, and this results in changes in blood pressure and heart rate [3]. Moreover, the hyperpolarization of sympathetic ganglia produced by 5-HT is caused by the activation of 5-HT_1A_ receptors in rats; these 5-HT_1A_ receptors were identified by using selective agonists and antagonists (see Table 1).

#### 1.3.3. Cardiac Effects of 5-HT

Central or i.v. administration of 5-HT may produce bradycardia and/or tachycardia, and the 5-HT receptors involved in these effects have been identified by using some of the agonists and antagonists shown in Table 1 [3,6].

Overall, two central 5-HT receptors regulate cardiovascular function: 5-HT_1A_ receptors (generally inhibiting the sympathetic drive) and 5-HT_2_ receptors (largely stimulating the sympathetic drive) [3,10,11]; some of the agonists and antagonists used to identify these receptors (with the inclusion and exclusion criteria described in Section 1.1) are shown in Table 1. Admittedly, central administration of 5-HT elicits complex and contradictory cardiac effects which depend on, among other factors, the species, the exact site of central application, the drug used, and the dose employed [3,10,11]. In contrast, the bradycardia or tachycardia produced by i.v. administration of 5-HT is more controllable and consistent (see below) in view of the implied simplicity of the procedure.

##### Bradycardia

I.v. administration of 5-HT in intact animals results in a pronounced and transient bradycardia that is abolished after ganglion blockade, vagotomy, atropine, spinal section, or 5-HT_3_ receptor antagonists [3,6]. This response involves the Bezold–Jarisch reflex, originating from the depolarization of afferent cardiac sensory neurons via activation of 5-HT_3_ receptors [3,6]. Furthermore, 5-HT can also produce bradycardia by (i) a cardiac sympatho-inhibition via activation of prejunctional 5-HT_1B_, 5-HT_1D_ and 5-HT_5A_ receptors in pithed rats [3,12,13]; or (ii) a cardiac vagal stimulation via activation of 5-HT_3_ receptors on parasympathetic ganglia and postganglionic vagal nerves in rabbits [3,6] (see Table 1 for pharmacological tools).

##### Tachycardia

I.v. administration of 5-HT in vagotomised animals induces a tachycardic effect that may be mediated by a wide variety of receptors/mechanisms, depending on the species and the experimental conditions [3,6]. These receptors/mechanisms include: (i) a tyramine-like action in spinal guinea pigs; (ii) direct stimulation of 5-HT_2A_ receptors on the cardiac pacemaker in reserpinized pithed rats; (iii) activation of 5-HT_3_ receptors on cardiac sympathetic neurons in the perfused heart of a rabbit, resulting in noradrenaline release and cardiac stimulation; (iv) activation of 5-HT_3_ receptors on a calcitonin gene-related peptide (CGRP)-containing sensory neurons in an isolated guinea pig atrium, resulting in CGRP release and cardiac stimulation; (v) direct stimulation of 5-HT_3_ receptors on a cardiac pacemaker in conscious dogs; (vi) direct stimulation of 5-HT_4_ receptors on a cardiac pacemaker in healthy anaesthetized pigs (which is also involved in the positive inotropic effects of 5-HT in isolated human atria and in rats with chronic heart failure); (vii) direct stimulation of 5-HT_7_ receptors on a cardiac pacemaker in spinal cats; and (viii) unidentified mechanisms in the isolated hearts of certain lamellibranch and gastropod species (including *Mercenaria mercenaria*, *Patella vulgata*, *Tapes watlingi*, *Helix aspersa*, *Aplysia* spp., etc.). These receptors were pharmacologically identified using selective agonists and antagonists for each type, and the inclusion and exclusion criteria explained in Section 1.1. (see Table 1).

#### 1.3.4. Vascular and Blood Pressure Effects of 5-HT

As explained in other reviews [3,7,8], i.v. administration of 5-HT results in a triphasic effect on arterial blood pressure, consisting of an initial transient vasodepressor effect followed by a vasopressor effect, and then a late long-lasting vasodepressor effect.

##### Initial Transient Vasodepressor Effect

This response results from an abrupt bradycardia (and the consequent decrease in cardiac output) following stimulation of 5-HT_3_ receptors on afferent cardiac vagal afferents (i.e., the Bezold–Jarisch reflex; see above and Table 1).

##### Vasopressor Effect

This effect (which varies quantitatively, depending on the species and the experimental conditions) involves the activation of vascular 5-HT_2_ receptors in resistance blood vessels (resulting in peripheral vasoconstriction). It is worth noting that a release of catecholamines by activation of 5-HT_2_ receptors in the adrenal medulla also plays a role in dogs, whereas activation of 5-HT_1B_ receptors produces vasoconstriction in cranial and carotid arteries in humans, pigs and dogs [3]. Interestingly, 5-HT_1B_ and 5-HT_2_ receptors elicit vasoconstriction in the internal carotid bed of anaesthetised dogs, while 5-HT directly activates, in vitro, α-adrenoceptors in rabbit ears and external carotid arteries [3]. Some of the agonists and antagonists used to identify these receptors (applying the inclusion and exclusion criteria defined in Section 1.1) are shown in Table 1.

##### Late Long-Lasting Vasodepressor Effect

This effect predominantly involves the activation of musculotropic 5-HT_7_ receptors [3,7,8], although several receptors/mechanisms may play a role, depending on the experimental conditions. These receptors/mechanisms may include:

*(i) Direct vasodilatation.* The direct vasodilatation to 5-HT involves 5-HT_7_ receptors in a wide variety of blood vessels under different experimental conditions [3,6,7,8]. Some of the agonists and antagonists used to identify these receptors (applying the aforementioned inclusion and exclusion criteria) are shown in Table 1. Moreover, in the blood vessels where 5-HT_7_ receptors produce vasodilatation and 5-HT_2_/5-HT_1B_ receptors produce vasoconstriction, the final effect of 5-HT would depend on the pre-existing vascular tone, the dose employed, and the proportions in which these receptors are distributed [3].

**Figure 1:**
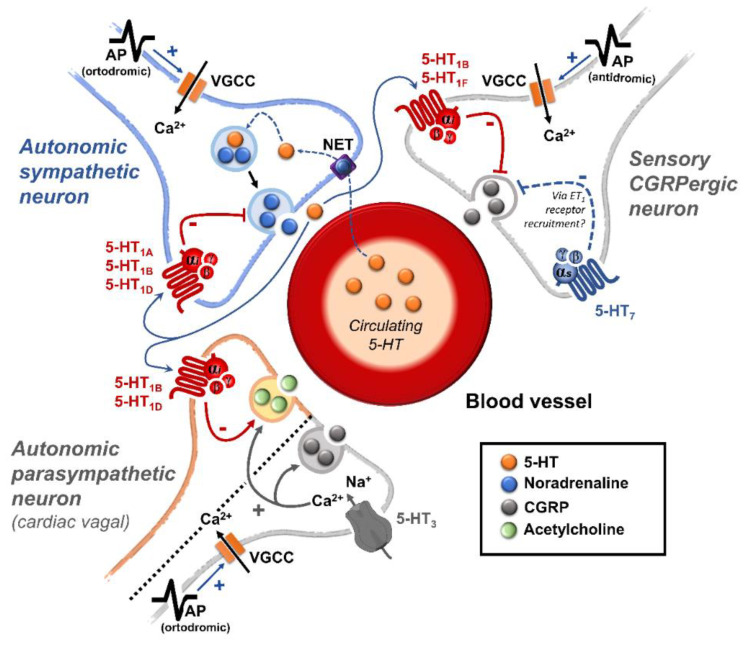
Prejunctional 5-HT receptors are involved in the inhibition of postganglionic autonomic and sensory CGRPergic function at the vascular level. Generally, 5-HT can inhibit the release of noradrenaline, acetylcholine, and CGRP via activation of the 5-HT_1_ receptor family (coupled to G*_i/o_* proteins; this figure shows the corresponding G_α/β/γ_ subunits). In the case of the parasympathetic outflow, activation of 5-HT_3_ (ligand-gated ion channel) receptors favours the release of acetylcholine. Furthermore, in sensory CGRPergic neurons, prejunctional activation of 5-HT_7_ receptors seems to recruit the endothelin system (via an unknown pathway), favouring the activation of the ET_1_ receptor and promoting inhibition of CGRP release. Interestingly, (i) in some isolated cases, activation of prejunctional 5-HT_3_ receptors on parasympathetic fibres facilitates the release of CGRP; and (ii) circulating 5-HT can be recaptured via NET, and subsequently vesiculated and released upon electrical stimulation of the sympathetic outflow. See text for details. AP: action potential; NET: noradrenaline transporters; VGCC: voltage-gated ion channels.

*(ii) Prejunctional inhibition of perivascular sympathetic neurons.* The prejunctional inhibition induced by 5-HT and related agonists on perivascular sympathetic neurons has been confirmed in vitro and in vivo in many blood vessels [3]. This vascular sympatho-inhibition, generally mediated by 5-HT_1_ receptors, may involve the 5-HT_1A_, 5-HT_1B_ and/or 5-HT_1D_ receptor subtypes, depending on the vascular bed under study, the species, and the experimental conditions [3]. Interestingly, sympatho-inhibitory 5-HT_7_ receptors could also be involved when rats are chronically pretreated with the 5-HT_2_ receptor antagonist sarpogrelate [3,14]. These receptors were pharmacologically identified by applying the inclusion and exclusion criteria explained in Section 1.1 (see Table 1 and Figure 1).

*(iii) Endothelium-dependent vasodilatation.* In isolated blood vessels of several species without a functional endothelium, the vasodilatation to 5-HT is attenuated, while the vasoconstriction is augmented [3]. This vasodilatation, involving endothelial release of nitric oxide (NO), is mainly mediated by 5-HT_1_ receptors [3]. Interestingly, in porcine blood vessels, the 5-HT-induced endothelium-dependent vasodilatation involves (i) 5-HT_1B/1D_ receptors in coronary arteries; or (ii) 5-HT_2B_ receptors in pulmonary arteries (see Table 1).

*(iv) Actions in the CNS.* Central administration of 5-HT may produce vasodepressor, vasopressor or biphasic effects, depending on the exact site of application, dose employed, depth of anaesthesia, the species used, etc. [3]. As previously reviewed [3,11], the cardiovascular regulation by central 5-HT neurons involves (i) 5-HT_1A_ receptors (associated with sympatho-inhibition, hypotension, and bradycardia); and (ii) 5-HT_2_ receptors (associated with sympatho-excitation and hypertension). Indeed, when directly applied in the CNS, 5-HT may produce both sympatho-inhibition and cardiac-vagal stimulation via 5-HT_1A_ receptors [10,15]. In fact, psychiatric conditions that involve alterations in the serotoninergic limbic components are usually accompanied by an autonomic imbalance; for example, posttraumatic stress disorder includes clinical manifestations such as cardiac arrhythmia, tachycardia, high blood pressure, etc. [16,17]. Moreover, anxiety correlates strongly with adrenaline levels in a positive direction [18], while aberrations in the autonomic nervous system (ANS) have been reported in patients with depression or other mood alterations [19]. Hence, central 5-HT is a powerful modulator of the ANS whose complex mechanisms fall beyond the scope of the present review. Interestingly, brain 5-HT can cross the blood–brain barrier via the 5-HT reuptake transporter (SERT) in endothelial cells and, consequently, can reach systemic circulation [20].

#### 1.3.5. Receptor-Independent Actions of 5-HT

Apart from the above cardiovascular effects of 5-HT mediated by 5-HT receptors, other studies suggest that 5-HT can also play cardiovascular (patho)physiological roles independent of 5-HT receptor activation [3]. For example, (i) rats pretreated with fluoxetine (a SERT inhibitor) were protected from monocrotaline-induced pulmonary hypertension [21]; and (ii) 5-HT uptake can “*serotonylate*” proteins by transglutaminase-2 [22], a mechanism involved in the mitogenic and profibrotic effects of 5-HT without receptor activation [23].

## 2. Peripheral Autonomic Nervous System and Prejunctional 5-HT Receptors

### 2.1. An Overview of the Peripheral Actions of 5-HT Regulating the Vascular Function

Although 5-HT modulates the ANS at the central level [3,10,11], presynaptic and pre-junctional mechanisms by which 5-HT controls perivascular cholinergic and adrenergic outflows are relevant. Indeed, mutant mice lacking the SERT gene showed increased plasma noradrenaline levels after immobilization [24]; this suggests that, during stressful situations, peripheral 5-HT reuptake may be an essential mechanism in the systemic catecholaminergic modulation by 5-HT. Nevertheless, acute and systemic administration of SERT inhibitors may produce sympathetic inhibition (mainly by central mechanisms) [25].

Certainly, both SERT and 5-HT receptors are expressed in rodent adrenal glands, particularly in chromaffin cells [26], and 5-HT is involved in the development of the adrenal medulla [27]. Moreover, the number of adrenal chromaffin cells in mice embryos seems to be controlled by 5-HT_3_ receptors expressed in their Schwann cell precursors [28]. Hence, 5-HT modulates adrenal chromaffin cells since their early development and, probably, during the rest of their lifetime.

Interestingly, when considering the distribution of SERTs in the adrenal chromaffin cells population, 5-HT seems to be strategically taken up by cells that exert an autocrine/paracrine modulation on the rest of chromaffin cells that release several vasocontractile mediators to the systemic circulation; these include adrenaline (~79%), noradrenaline (~18%), and other mediators (~1–3%) during a sympathetic fight/flight situation induced by fear, stress, exercise, or conflict [26,29]. In this manner, the adrenal chromaffin release is controlled both neurogenically (by the ANS) and non-neurogenically (by several mediators, including 5-HT) [26,29].

On the other hand, it is noteworthy that adrenal chromaffin cells do not synthesize 5-HT by themselves [30,31], but they can take up 5-HT via their high expression of SERTs [26,31]. Moreover, activation of 5-HT_1A_ receptors decreased adrenal chromaffin release [19,30]. Hence, 5-HT may act as a neuroendocrine tool to modulate (negatively) catecholamines release after stressful events via 5-HT_1_ receptors. In addition, the autonomic control of perivascular sympathetic nerves is strategically organized to exert a local modulation of blood vessel sections, or even complete vascular beds [32]; hence, these sympathetic nerves form a complex varicose network that surrounds the blood vessels at the level of the adventitia layer in close proximity with the smooth muscle cells. However, neurotransmitters can diffuse and reach the endothelium [33]; this opens the possibility for a highly specific modulation by 5-HT of each blood vessel layer, namely, tunica intima, tunica media, and tunica externa (also called tunica adventitia).

The parasympathetic branch of the ANS innervates only cerebral vascular beds, whereas it does not innervate peripheral resistance blood vessels [34,35]. Particularly, intracerebral posterior blood vessels are richly innervated by parasympathetic fibres that seem to exert an essential control of blood flow in the polygon of Willis [36]. In peripheral blood vessels, vagal parasympathetic molecules (mainly acetylcholine) may be released systemically and reach the endothelium, exerting vasorelaxant neuroendocrine actions [33]. In short, both sympathetic and vagal parasympathetic varicosities express 5-HT receptors [37,38]. Thus, 5-HT may modulate sympathetic and parasympathetic perivascular nerves, and exert direct vascular actions [39,40], as described in Section 1.

### 2.2. The Role of Prejunctional 5-HT Receptors

There are several sources of 5-HT that may contribute to the modulation of perivascular autonomic and sensory nerve terminals; these include (i) the systemic circulation, where 5-HT is transported via blood platelets and released upon activation [41,42]; (ii) chromaffin cells of the adrenal medulla [26,30]; (iii) enterochromaffin gastrointestinal cells [43]; (iv) a subgroup of trigeminal C-fibres which store 5-HT [44]; and (v) cortical terminals from raphe neurons [20].

In the parasympathetic branch, the sphenopalatine ganglion (SPG) positively regulates cerebral blood flow; interestingly, more than 96% of the SPG body cells express 5-HT receptors [45]. Hence, 5-HT may be seen as a ubiquitous autonomic modulator.

5-HT_1/2/3_ receptors are highly active during motor, sensory, and autonomic neuron development [46]. Thus, these receptors may maintain homeostatic functions in the motor, sensory, and autonomic neurons of the developed organism. According to their transduction systems, 5-HT_1_ receptors are mainly involved in sympathetic inhibition, whereas 5-HT_2_ and 5-HT_3_ receptors may facilitate parasympathetic outflow (see below).

#### 2.2.1. The 5-HT Receptors Inhibiting the Autonomic Outflow

Apart from its central sites of action, 5-HT can inhibit the tachycardia induced by sympathetic electrical stimulation, but not the one induced by exogenous noradrenaline [47]. This finding revealed the existence of a 5-HT-induced cardiac sympatho-inhibition at the prejunctional level (Figure 1).

5-HT_1_ receptors are widely expressed in perivascular and cardiac sympathetic nerve endings; their activation is linked to cardiovascular sympathetic inhibition [48,49,50,51]. Specifically, selective stimulation of 5-HT_1A_, 5-HT_1B_, and 5-HT_1D_ receptors produced inhibition of the sympathetic vasopressor outflow in pithed rats [38,52]. This is a useful experimental model to pharmacologically study the prejunctional modulation of sympathetic nerve endings, since the CNS in not functional and, consequently, central compensatory cardiovascular reflexes are excluded [3]. Furthermore, in vitro experiments in the human atrium have shown that sympathetic nerves express 5-HT_1D_ receptors, which mediate sympathetic inhibition [53]. Similarly, in pithed rats, the sympathetic cardioaccelerator outflow is inhibited by 5-HT_1B/1D_ receptor activation [12].

#### 2.2.2. The 5-HT Receptors as Facilitators of the Autonomic Outflow

In pithed rats, the bradycardia induced by vagal electrical stimulation may be increased by 5-HT during the blockade of 5-HT_1/2_ receptors and by selective 5-HT_3_ receptor agonists [54]. In contrast, activation of 5-HT_2_ receptors inhibited this bradycardia induced by vagal electrical stimulation [54]. These findings suggest a dual role for 5-HT receptors in the cardiac parasympathetic outflow. Interestingly, in cerebral blood vessels, most SPG parasympathetic neurons (i) highly express 5-HT_3A_ > 5-HT_3B_ receptors; (ii) slightly express 5-HT_2B_ > 5-HT_2A_ > 5-HT_1B_ receptors; and (iii) practically lack the expression of 5-HT_1A_, 5-HT_1D_, 5-HT_1F_, 5-HT_2C_, 5-HT_4_, 5-HT_5A_, 5-HT_5B_, 5-HT_6_ and 5-HT_7_ receptors [45]. In view that the 5-HT_3_ receptor forms a ligand-gated Na^+^/K^+^ channel, its parasympathetic expression in SPG neurons leads to a release of acetylcholine, NO, and vasoactive intestinal polypeptide (VIP), which, in turn, results in vasodilatation [55]. On the other hand, it remains unclear whether major cerebral blood vessels are rich in 5-HT receptors [56].

### 2.3. Clinical Relevance and Therapeutic Potential

The serotoninergic negative modulation of sympathetic cardiovascular activity by 5-HT_1A/1B/1D_ receptors may be achieved endogenously through platelet activation by catecholamines [42]. Indeed, a recent clinical study exposed 79 healthy male and female volunteers to tryptophan enhancement, and 85 others to tryptophan depletion conditions, to analyse adrenaline and noradrenaline plasma levels [57]. Participants from the tryptophan enhancement condition showed a clear increment in plasma adrenaline, while noradrenaline decreased. Interestingly, this depletion condition slightly increased adrenaline and noradrenaline levels compared with baseline [57], suggesting that some preclinical findings are also observed clinically. Hence, 5-HT_1_ receptors on perivascular fibres represent therapeutic targets to decrease sympathetic noradrenaline release.

On the other hand, a metanalysis with cancer and cancer-depressed patients [58] concluded that management of stress-linked-emotions (which include serotoninergic alterations) is a crucial element to (i) prevent comorbidities related to disruption of endocrine and autonomic (sympathetic) nervous system homeostasis; and (ii) improve survival time in these patients. Hence, stabilizing 5-HT levels may be a strategy to prevent autonomic disorders as comorbidities in diseases with associated high emotional stress.

It is important to keep in mind that some blood vessels (e.g., those from the coronary and carotid vascular beds) express the 5-HT_1_ receptor in the smooth muscle layer, whose activation produces vasoconstriction [59]. These receptors are directly and/or indirectly involved in the pharmacotherapy of migraine, and some of the prophylactic (e.g., methysergide) and acute (e.g., triptans and ergots) antimigraine drugs interact with these receptors [60,61,62]. Hence, as a well-founded concern, direct vascular effects should be considered in any strategy that modifies 5-HT levels.

## 3. Sensory CGRPergic Perivascular Nerves and Prejunctional 5-HT Receptors

### 3.1. The Sensory Perivascular CGRPergic Neurons as an Intrinsic Modulator of Vascular Tone

In general, CGRP is a potent vasodilator that can be released by capsaicin [63]; hence, CGRP release is associated with the activation of TRPV1 receptors on sensory nerves [64,65]. Nevertheless, the role of other TRP ion channels (e.g., TRPA1, TRPM3) located on nociceptors that induce the release of CGRP, has also been documented [66]. It is noteworthy that sensory nerves, which originate from the spinal cord [67], can exert (i) afferent actions [67]; and (ii) efferent actions via local (axonal) or central (dorsal root) reflexes [9]. In contrast to the efferent autonomic perivascular innervation from the spinal ventral horn, the sensory afferent fibres arrive at the spinal dorsal horn conveying information from the periphery to the spinal cord [9].

The relevance of the sensory nervous system (particularly CGRP release) as an intrinsic modulator of vascular tone was elegantly demonstrated by a series of in situ and in vivo experiments led by the group of Kawasaki in the early 90s. Indeed, they showed that, after pharmacological blockade of autonomic function, electrical stimulation of perivascular sensory nerves resulted in a vasodilator action mediated by CGRP release (blocked by CGRP_(8–37)_, a CGRP receptor antagonist), which was insensitive to blockade of β-adrenergic, muscarinic and histaminergic receptors [68,69,70,71]. More recently, our group has shown in pithed rats that, after CGRP receptor blockade with olcegepant, not only are the neurogenic and non-neurogenic vasodepressor responses to CGRP precluded, but a potentiation of the noradrenergic vasopressor responses is also unmasked [72]. Together, these data demonstrate that selective stimulation of perivascular sensory nerves results in CGRP release at the prejunctional level, activating CGRP receptors and evoking vasodilation. Current data strongly support the notion that CGRPergic sensory transmission modulates vascular tone via smooth muscle or endothelial mechanisms [73,74].

At the prejunctional level, several mechanisms have been reported to impact the sensory release of CGRP. One of the first lines of evidence suggesting that prejunctional heteroreceptors in sensory nerves modulate CGRP release was observed in experiments performed in the mesenteric vascular beds [69]. Briefly, Kawasaki et al. [69] showed that the vasodilation induced by periarterial nerve stimulation is smaller in vascular beds precontracted with noradrenaline (the endogenous ligand; non-selective α_1/2_- and β-adrenergic agonist) than in those precontracted with methoxamine (a selective α_1_-adrenoceptor agonist); this finding correlated with activation of α_2_-adrenoceptor activation [68]. These data suggest that the sympathetic perivascular outflow induces a direct vasoconstrictor effect mediated by vascular activation of α_1/2_-adrenoceptors, and an indirect action by inhibiting the vasodilator function of sensory perivascular fibres. Furthermore, since α_2_-adrenoceptors are divided into three functional subtypes (α_2A/2B/2C_-), further pharmacological analysis in pithed rats showed that a fine-tuning of the perivascular sensory release of CGRP at the systemic level exists by selective activation of α_2A/2C_-adrenoceptors [75]. In this regard, several other prejunctional heteroreceptors facilitating (e.g., TRPV_1_) or inhibiting (e.g., µ-opioid, D_2_-like, CB_1_, H_3,_ P2Y_1/13_, and 5-HT_1_ receptors) CGRPergic neurovascular transmission have been described (for references see [9]).

It is worth noting that the potential relevance of serotonergic transmission modulating the perivascular sensory CGRPergic outflow has been established in the last 15 years [76,77]. In the case of 5-HT receptors modulating perivascular CGRPergic transmission, special attention has been paid in the context of migraine pathophysiology and pharmacotherapy. Indeed, triptans such as sumatriptan, which is a 5-HT_1B/1D/1F_ receptor agonist considered the gold standard in acute migraine treatment, aborts migraine attacks by producing (i) direct vasoconstriction of intracranial and extracranial arteries; and (ii) inhibition of CGRP release at the trigeminal level and on perivascular sensory nerves [60,78].

### 3.2. Prejunctional 5-HT Receptors as Inhibitors of the Perivascular Sensory CGRPergic Outflow

As mentioned above, triptans and ergots (agonists at 5-HT_1_ receptors) can prejunctionally inhibit the trigeminal release of CGRP [79,80]. Indeed, the first evidence about the role of 5-HT_1_ receptors as inhibitors of CGRPergic transmission derived from pharmacological research on the mechanisms involved in the therapeutic effects of acute antimigraine drugs [81,82,83,84]. Admittedly, the discussion on the relevance of serotonergic mechanisms modulating CGRPergic outflow in the context of antimigraine therapy (i.e., at trigeminovascular level) falls beyond the scope of the present review, and several excellent reviews on this topic have been published elsewhere (see refs. [60,79,85,86,87]).

Considering that triptans and ergots are associated with cardiovascular side effects [60], a study in pithed rats demonstrated that acute (rather than prophylactic) antimigraine drugs are capable of inhibiting the perivascular sensory CGRPergic outflow at the systemic level, via prejunctional mechanisms [88]. Specifically, the pithed rat model was used to analyse vascular and prejunctional mechanisms excluding the influence of any central compensatory reflex mechanisms. Under these experimental conditions, in animals infused with hexamethonium (a sympathetic ganglionic blocker) and methoxamine (an α_1_-adrenoceptor agonist to induce sustained systemic vasoconstriction), the treatment with sumatriptan, ergotamine, or dihydroergotamine inhibited the vasodepressor responses elicited by electrical stimulation of the T_9_–T_12_ spinal cord segments (an effect associated with inhibition of CGRP release from perivascular sensory nerves [88]).

The above data strongly support the hypothesis that 5-HT receptors on perivascular sensory nerve endings modulate CGRP release in the vascular system (such as at the trigeminovascular level) (Figure 1). Certainly, molecular biology evidence at the dorsal root ganglion level has suggested that mRNA expression correlates with 5-HT_1B_ and 5-HT_1F_, but not with 5-HT_1A_ or 5-HT_1D_, receptors [89]. In this regard, further functional pharmacological experiments using the pithed rat model showed that the selective 5-HT_1B_ receptor agonist, CP-93,129, selectively inhibits the neurogenic CGRPergic vasodepressor responses via prejunctional sensory mechanisms [77]. Likewise, some data suggest that trigeminal activation of prejunctional 5-HT_1B_ receptors (by sumatriptan or donitriptan) inhibits the external carotid vasodilation induced by capsaicin [90,91], highlighting the relevance of this receptor subtype in the modulation of CGRP release. Furthermore, as discussed by Rubio-Beltrán et al. [92], since 5-HT_1F_ receptors have been found on sensory nerves, the role of these receptors in the modulation of CGRP release is suggested. Indeed, lasmiditan (a selective 5-HT_1F_ receptor agonist) can prejunctionally inhibit CGRP release not only at the central (trigeminal) level, but also at the peripheral (meninges) level [80].

In view that sumatriptan is a non-selective 5-HT_1A/1B/1D/1F_ receptor agonist, the role of these receptor subtypes was also analysed in the inhibition of the vasodepressor sensory CGRPergic outflow in pithed rats [76]. The data using selective agonists and antagonists for each 5-HT_1_ receptor subtype (see Table 1) (i) corroborated the relevance of 5-HT_1B_ receptors, and further showed that activation of prejunctional 5-HT_1F_ receptors inhibited CGRP release; and (ii) excluded the role of 5-HT_1A_ and 5-HT_1D_ receptors [76]. It is noteworthy that prejunctional 5-HT_1D_ receptors have also been suggested to inhibit the trigeminal release of CGRP [93]; however, PNU-142633 (a selective 5-HT_1D_ receptor agonist; see Table 1) was not effective in the acute treatment of migraine [94], a neurovascular disorder where trigeminal release of CGRP plays a key role [85,86,87].

The ergots ergotamine and dihydroergotamine can also inhibit the perivascular sensory CGRPergic outflow [60]. Nevertheless, their pharmacology is much more complex, since these compounds display an affinity for all 5-HT (except 5-HT_3_) receptors [3,4,5] and also interact with dopaminergic and noradrenergic receptors [3]. Indeed, a detailed pharmacological analysis showed that, apart from prejunctional 5-HT_1B/1F_ receptors, these ergots activate prejunctional D_2_-like and α_2_-adrenergic receptors to inhibit the vasodepressor sensory CGRPergic outflow [60,95].

Regarding transductional mechanisms, the 5-HT_1_ receptor family is coupled to G*_i/o_* proteins [1] which, in turn, (i) via the G_α_ subunit reduce the activity of adenylate cyclase, diminishing intracellular cAMP levels and consequently inhibiting the activity of protein kinase A; and (ii) via the G_β/γ_ subunits increase the activity of K^+^ channels. Both mechanisms are intrinsically associated with the inhibition of neurotransmitter release [96]. From this perspective, the fact that AS-19 (a 5-HT_7_ receptor agonist; Table 1) activated prejunctional 5-HT_7_ receptors to inhibit the rat vasodepressor sensory CGRPergic outflow was counterintuitive [97] since this receptor is positively coupled to G*_s_* proteins [1]. Hence, one would have expected facilitation rather than inhibition of this sensory CGRPergic outflow. Nonetheless, the possibility exists that this 5-HT_7_ receptor-induced sensory inhibition may involve (i) an ATP-dependent K^+^ channel-mediated hyperpolarization sensitive to glibenclamide [97], as reported for the 5-HT-induced inhibition of the contractile and electrical activities in the guinea pig mesenteric bed [98]; and (ii) the endothelin pathway, as this response was blocked by sulfisoxazole [97], an endothelin ET_A_ receptor antagonist [99]. Indeed, endothelin-1 can inhibit the neuroeffector transmission in smooth muscle [100], as well as the capsaicin-induced CGRP release [101]. Hence, the prejunctional 5-HT_7_ receptor seems to promote endothelin-1 secretion, which inhibits CGRP release.

Moreover, activation of the 5-HT_7_ receptor at the spinal cord level results in an antinociceptive action whereas, at the peripheral level, it enhances capsaicin-induced sensitization [102]. Clearly, the location of this receptor is the determining factor in its effects.

Interestingly, molecular expression analysis of 5-HT receptors in dorsal root ganglion neurons showed that, apart from 5-HT_1B_, 5-HT_1F,_ and 5-HT_7_ receptors, 5-HT_2A_, 5-HT_2C_, 5-HT_3_, 5-HT_5A_, 5-HT_5B_, and 5-HT_6_ receptors can also be found in this type of cells [89,103,104]. Although the functional role of the latter receptor (sub)types in sensory vascular neurotransmission has not yet been reported, experiments exploring nociception showed that activation of 5-HT_2A_ or 5-HT_4_ receptors enhances CGRPergic transmission [105,106], while activation of 5-HT_5A_ receptors have the opposite effect [107]. Furthermore, in guinea pig isolated cardiac atria, 5-HT favours CGRP release via sensory 5-HT_3_ receptor activation, leading to a positive inotropic response [108]. However, it must be highlighted that these findings do not necessarily imply that similar results can be obtained at the vascular sensory neuroeffector level, as illustrated above with the case of the 5-HT_7_ receptor.

### 3.3. Clinical Relevance

Apart from the well-established therapeutic relevance of acute antimigraine serotonergic drugs, which inhibit trigeminal CGRPergic transmission by 5-HT_1B/1D/1F_ receptor activation [92], little attention has been paid to the interaction between 5-HT and the perivascular sensory nerves modulating systemic vascular responses (i.e., changes in arterial blood pressure). Admittedly, this is partly because there is no consensus on the pivotal role of CGRP in maintaining blood pressure [73,109]. In addition, since the pharmacology of serotonergic transmission is complex at the peripheral and central levels, the (cardio)vascular effects resulting from activation of the different 5-HT receptors at both levels are hard to explain [3,7,8].

Regarding perivascular CGRPergic transmission on the systemic vasculature, some findings seem to exclude the relevance of this neuropeptide in regulating blood pressure, since acute CGRP receptor blockade in anaesthetized rats does not significantly impact blood pressure levels [74,109]. Accordingly, resting blood pressure is not affected in transgenic mice lacking the CGRP receptor [110]; conversely, continuous recording of blood pressure (in CGRP receptor KO mice) showed that this parameter is globally increased by an enhancement of the sympathetic autonomic function [111]. Indeed, in pithed rats (in which the CNS in not functional), acute pharmacological blockade of the CGRP receptor with olcegepant not only inhibits the vasodepressor sensory CGRPergic outflow, but also enhanced the sympathetic vasopressor responses [72]. These data may imply that continuous blockade of CGRPergic vascular transmission with olcegepant (or any other CGRP antagonist) could favour a hypertensive state [72]. Although this effect was apparently absent in clinical trials [112], real-world studies now suggest that the use of CGRP (receptor) blocking medications may increase blood pressure [113]. As elsewhere discussed [73,114,115], CGRP may play a physiological protective role in the cardiovascular system; nevertheless, the relevance of CGRPergic transmission in blood pressure regulation is only unmasked in cardiovascular pathologies [116].

Certainly, a decrease in CGRP levels has been observed in spontaneously hypertensive rats and in humans with essential hypertension [117,118]; in addition, it has been suggested that a diminution of the perivascular CGRPergic innervation may play a role in the development of this pathology [119]. Furthermore, within the bounds of 5-HT receptors, it is to be highlighted that, globally, 5-HT produces vasopressor responses by activation of vascular 5-HT_2A_ receptors [3,8]; however, under vascular damage conditions (e.g., in hypertension), the vasculature is more sensitive to 5-HT to cause contraction [8]. Thus, apart from an enhanced 5-HT-induced vasoconstriction in hypertensive subjects [8], we suggest that the release of CGRP in these subjects may be diminished by activation of prejunctional 5-HT_1B/1F_ and 5-HT_7_ receptors, favouring a pro-hypertensive state.

## 4. Perspectives and Some Future Directions 

Physiologically, blood pressure is regulated by changes in peripheral vascular tone (caused by resistance blood vessels) and cardiac output, and these parameters are homeostatically maintained by neuronal, humoral, and local mechanisms [120]. When considering the neurovascular junction, it is well known that vascular tone is modulated by (i) autonomic sympathetic nerves, which produce vasoconstriction by noradrenaline release [120,121]; and (ii) primary sensory nerves, which produce vasodilatation by neuropeptides release, mainly CGRP [69,70,122,123,124,125]. Several mechanisms exist at the neurovascular junction to modulate the neuronal outflow to the blood vessels; one of these mechanisms is the serotonergic transmission. Certainly, 5-HT can generally modulate the autonomic and sensory outflows via prejunctional receptors (see Figure 1) to regulate blood pressure.

From a global perspective, 5-HT is an amphibaric agent, and its actions on haemodynamic parameters are complex, depending on the experimental conditions [3,7]. This may be explained in terms of the numerous sites of action to 5-HT, which include: (i) the CNS; (ii) autonomic ganglia; (iii) perivascular autonomic and sensory nerve terminals; (iv) endothelial cells; (v) smooth muscle cells; and (vi) the heart [3,37,38,46,126,127]. Consequently, rather than assigning a single cardiovascular function to 5-HT, it is clear that 5-HT exerts multiple cardiovascular actions, which may be further complicated by pathophysiological states including, but not limited to, hypertension [3,7], depression [128], and pain [128]. Thus, when studying the effects of 5-HT, it is imperative to be open-minded about its possible pleiotropic actions.

On this basis, future research should focus, among other approaches, on the significance of interspecies differences, the (patho)physiological conditions that may affect the function of 5-HT and its receptors/transporters, as well as interindividual differences caused by gender, ethnic background, body mass index, age, etc. For example, 17β-oestradiol may modulate the SERT and 5-HT metabolism in the brain [129], and it is well known that the correct function of the serotonergic system critically depends on the function of the SERT [130]; hence, this finding may be relevant in perimenopausal women.

Furthermore, together with the seminal discovery that 5-HT is a vasoconstrictor [131], the fact that plasma 5-HT levels are increased in hypertension favoured the hypothesis about the hypertensive action of 5-HT [132]. However, a chronic 5-HT infusion induced a fall in blood pressure [133], probably via an increase in endothelial NOs system activity [134]. This effect is clearly dependent on SERT function, as SERT knock-out mice are less prone to the chronic 5-HT-induced drop in blood pressure [134]. Interestingly, some clinical studies have shown that chronic inhibition of 5-HT reuptake by SERT inhibitors reduced the risk of a myocardial infarction [135], but an increase in blood pressure was recorded at night [136]. In this regard, in dogs, chronic oral treatment with fluoxetine does not affect mean arterial blood pressure (measured at diurnal time), but the capsaicin-induced trigeminal CGRP release (resulting in vasodilatation) is diminished at the craniovascular (external carotid) level [137]; this finding may help explain the mechanism of action of some SERT inhibitors to treat migraine.

Moreover, other pathophysiological states (apart from hypertension, depression and pain) may affect the actions of the serotonergic system, as exemplified by a recent study describing that the SERT is negatively associated with body mass index after glucose loading [138]; this finding highlights the importance of a holistic approach, taking factors such as body weight and/or obesity into account when investigating the serotonergic system.

In addition to the above differential role of SERT in various conditions, the 5-HT receptor function may also have specific relevance in several circumstances. For example, the 5-HT_1F_ receptor has been described to promote, in rodents, mitochondrial biogenesis and recovery from acute kidney injury [139], as well as spinal cord injury [140], which might potentially also differ between sexes [140]. With these preclinical findings, it would be tempting to suggest in a clinical setting (including medical emergencies) that patients with kidney and spinal cord injuries could be treated with the selective 5-HT_1F_ receptor agonist lasmiditan; this is a Federal Drug Administration-approved antimigraine drug that prejunctionally inhibits the trigeminal release of CGRP [80,92].

## 5. Conclusions

As evident from the present review, in addition to the pathophysiological relevance of the SERT in the receptor-independent intracellular actions of 5-HT [23], this monoamine induces a plethora of complex, and sometimes opposing, actions in the cardiovascular system. These cardiovascular actions of 5-HT may be even further complicated by pathophysiological states [3,7,9,18,19,128], and also by 5-HT receptor intracellular signalling (i.e., the impact of biased agonists) [141]. Admittedly, it is challenging to unravel the effects of these conditions on the functioning of 5-HT and its receptors as well as transporters. Despite its complex action, research on human differentiated tissues obtained from induced pluripotent stem cells (iPSCs) may be a valuable tool for the study of rare diseases and the influence of different (culture) conditions. Because of the many modulating roles of 5-HT in other systems, targeting specific 5-HT receptors may provide valuable novel therapeutic avenues, besides its currently known therapeutic applications [1,3,4,5,6,7,8]. Accordingly, further understanding of the actions of 5-HT under different conditions will provide new insights and therapeutic treatment possibilities, with the serotonergic system as a pharmacological target.

## Figures and Tables

**Table 1:** Classification of 5-HT receptors ^a^.

5-HT Receptor	Receptor Subtype	Agonists	Antagonists	Some Functions	Canonical Transduction System
5-HT_1_	5-HT_1A_	8-OH-DPAT	WAY 100635	Central hypotension	*G-protein coupled receptor (G_i_)* 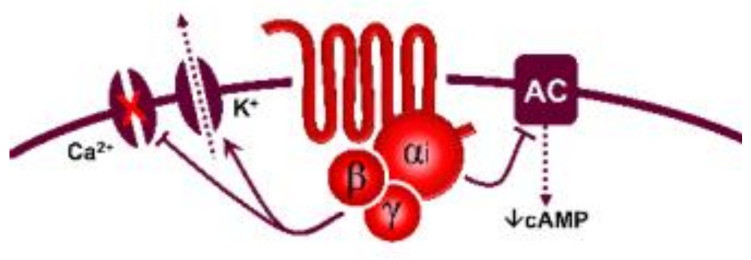
	5-HT_1B_	Sumatriptan CP-93,129 (rodents)	SB224289	Vasoconstriction, sympatho-inhibition	
	5-HT_1D_	PNU-109291 PNU-142633	BRL15572	Autoreceptor, sympatho-inhibition	
	5-HT_1e_ *	5-HT >> 5-CT, LY334370	Methiothepin (non-selective)	Unknown	
	5-HT_F_	LY344864, lasmiditan, LY334370	Methysergide (non-selective)	(−) Trigeminal system	
5-HT_5_	5-HT_5A_	5-HT, ergotamine	SB699551	Cardiac sympatho-inhibition in rats	
	5-HT_5b_ *	5-CT (non-selective)	Unknown	Unknown	
5-HT_4_	-	Renzapride, BIMU8, ML10302, SC53116	GR 113808, SB204070	(+) Neuronal activity, vasodilatation, tachycardia in pigs and humans	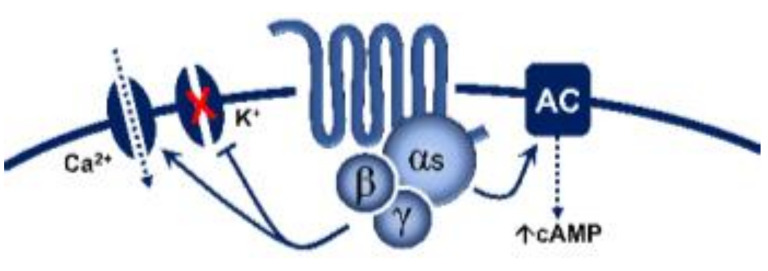 *G-protein coupled receptor (G_s_)*
5-HT_6_	-	5-MeO-T ≥ 5-HT SB357134, SB271046	Ro 630563	Memory, not involved in cardiovascular regulation	
5-HT_7_	-	5-CT>>5-HT AS-19	SB269970, SB258719	Circadian rhythm, vasodila- tation, tachycardia in cats	
5-HT_2_	5-HT_2A_	DOI, DOB α-methyl-5-HT	MDL100907 Ketanserin	Vasoconstriction, plateletaggregation	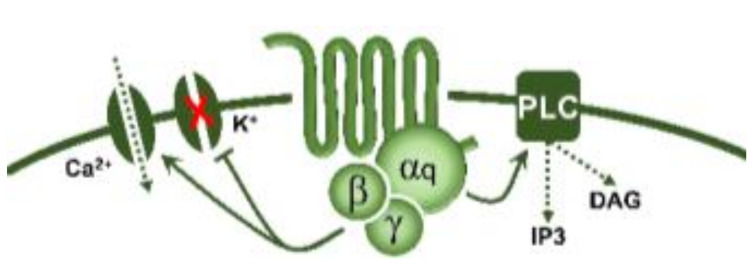 *G-protein coupled receptor (G_q_)*
	5-HT_2B_	DOI, BW723C86 α-methyl-5-HT	SB204741 RS-127445	Vasoconstriction, release of NO	
	5-HT_2C_	DOI, Ro 60-0175 α-methyl-5-HT	SB242084 RS-102221	CSF production	
5-HT_3_	Pentameric ion channel **	Phenylbiguanide 2-methyl-5-HT	Tropisetron, Granisetron MDL-72222	(+) Neuronal activity, reflex bradycardia	*Ligand-gated ion channel* 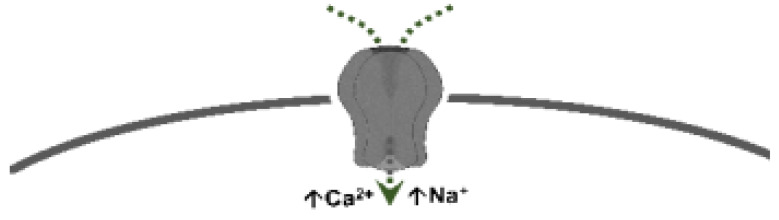

Modified from Villalón [3]. AS-19, (2*S*)-(+)-5-(1,3,5-Trimethylpyrazol-4-yl)-2-(dimethylamino)tetralin; CNS, central nervous system; CSF, cerebrospinal fluid; LSD, lysergic acid diethylamide; 5-MeOT, 5-methoxytryptamine; 5-CT, 5-carboxamidotryptamine; DOI, 1-(2,5-dimethoxy-4-iodophenyl)-2-aminopropane; NO, nitric oxide; (−), inhibits; (+), stimulates. * Lowercase is used to denote a receptor with unknown functional roles in native cells or tissues. ** Five known subunits have been described (5-HT_3A_–5-HT_3E_) forming homomeric or heteromeric complexes. At least two subunits of 5-HT_3A_ type are required to form a functional ion channel. ^a^ The pharmacological profile of each 5-HT receptor type is identified by applying inclusion and exclusion criteria, as explained in Section 1.1.

## Data Availability

The articles cited in this paper are available on PubMed^®^, UptoDate^®^, and Cochrane^®^.
